# The complete mitochondrial genome and phylogenetic analysis of *Festuca pratensis*

**DOI:** 10.3389/fpls.2026.1850006

**Published:** 2026-05-13

**Authors:** Wenlong Gong, Chunyu Tian, Yanting Yang, Lemeng Liu, Yumei Feng, Fengdan Wang, Zhiyong Li, Zinian Wu

**Affiliations:** 1 Institute of Grassland Research, Chinese Academy of Agricultural Sciences, Hohhot, China; 2 Key Laboratory of Grassland Resources and Utilization of Ministry of Agriculture, Hohhot, China

**Keywords:** complete mitochondrial genome, *Festuca pratensis*, phylogenetic analysis, RNA editing, synteny

## Abstract

**Introduction:**

*Festuca pratensis* is a perennial herb of *Festuca* in Poaceae. It has become an important forage and ecological restoration grass species due to its strong stress resistance and high nutritional value. Limited by the complexity of the forage grasses genome, the number of mitochondrial genome assembly in this genus is limited, which seriously restricts the research process of early mitochondrial genome evolution and key gene mining.

**Methods:**

We assembled and annotated the first high-quality mitochondrial genome of *Festuca pratensis* using third-generation and second-generation sequencing technology. The genome structure, sequence characteristics, evolutionary selection pressure and genetic relationship of *Festuca pratensis* mitochondrial genome were analyzed.

**Results and discussion:**

The mitochondrial genome has a multichromosomal structure with a total length of 449,613 bp. A total of 51 genes were detected, including 14 core genes, 15 tRNA genes, 19 variable genes and 3 rRNA genes. Three types of repetitive sequences were detected, including 121 simple repetitive sequences, 12 tandem repetitive sequences and 112 scattered repetitive sequences. A total of 9,810 codons encoding 33 PCGs were detected, and the probability of codon preference and avoidance was 50% and 47%, respectively. A total of 454 effective RNA editing sites were detected, distributed on 30 PCGs with all base change types C-U. 84 inter-genome transfer fragments were identified, and four complete transfer DNA fragments were *rps12*, *trnS-UGA*, *trnM-CAU* and *trnN-GUU*, respectively. The *rps3* gene showed positive selection (Ka/Ks > 1) in 36 (90%) of 40 species, indicating that it may be a functional gene adapted to rapid evolution. The Pi values of 33 genes ranged from 0 to 0.16277. Phylogenetic analysis divided 43 species into 6 categories. Combined with the results of collinearity, it was found that *Festuca pratensis* had the closest genetic relationship with *Lolium perenne*. In this study, the first high-quality mitochondrial genome of *Festuca pratensis* was assembled, which provided key high-quality reference data for subsequent organelle genome evolution, stress-resistant functional gene mining and germplasm resource identification of related species.

## 1 Introduction

*Festuca pratensis* is a perennial herb belonging to *Festuca* of Poaceae (Kopecký et al., 2010; Roderick et al., 2003), which distributed in Europe and southwest Asia. It is mainly cultivated in Jilin, Beijing, Qinghai and other provinces in China. It often grows on slopes, valleys and canals at an altitude of 700-2,800 meters (Fjellheim and Rognli, 2005; Alm et al., 2011). *Festuca pratensis* is widely distributed in grassland because of its strong drought resistance, salt tolerance and regeneration ability. Due to its high grass yield, good palatability and rich nutritional value, it has become the main source of forage for livestock and hay feed in winter (McLeod et al., 2001; Leśniewska et al., 2001). At the same time, the characteristics of *Festuca pratensis*, such as soft and spineless, long green period and trampling resistance, also make it play an important role in lawn construction and saline-alkali land restoration (Soleimani et al., 2010). There are also studies on the utilization of *Festuca* and *Lolium perenne* hybrid breeding high yield and strong resistance new varieties, which reflected its rich genetic diversity as breeding chassis materials (Majka et al., 2018; Pašakinskienė, 2023). However, the current research on *Festuca pratensis* mainly focuses on cultivation, water and fertilizer management, nutritional components and molecular markers (Samy et al., 2020; Majka et al., 2019; Kavka et al., 2024; Laihonen et al., 2022). In order to obtain complete and accurate genetic sequence and functional element information, it is necessary to excavate functional genes related to fertility, stress resistance, growth and yield, and to study its complete mitochondrial genomics.

Herbaceous plants are mostly perennial cross-pollinated plants. Their genomes are often characterized by high heterozygosity, complex ploidy and high proportion of repetitive sequences. The assembly of their nuclear genomes is often difficult, long cycle and high cost. At present, only some grasses have made progress (Chen et al., 2023; Ma et al., 2021; Feuillet and Keller, 2002; International Brachypodium Initiative, 2010). Plant mitochondria are semi-autonomous organelles with maternal genetic materials, which are useful tools for research the origin, genetic diversity and phylogeny of species (Soe et al., 2025; Wang et al., 2025). The genome structure of plant mitochondria is relatively conserved, the sequence length is moderate and the evolutionary signal is clear (Logan, 2006). With the rapid development of modern molecular biology, it is possible to obtain high-quality mitochondrial genomes of complex species using second-generation and third-generation sequencing techniques (Rose, 2021; Tang et al., 2024; Duminil and Besnard, 2021). Mitochondrial genomes often show significant structural diversity (circular, linear, branched, or mixed forms), and usually their sequences evolve slower than nuclear genomes and chloroplast genomes, but their DNA rearrangement rates are significantly higher than chloroplast DNA. The widespread post-transcriptional RNA editing can regulate the function and expression of genes and proteins (Forner, 2025). At present, important progress has been made in species classification, phylogenetic evolution, cytoplasmic male sterility mechanism, and nuclear-cytoplasmic interaction research (Arimura and Nakazato, 2024; Gualberto et al., 2014; Chevigny et al., 2020).

The number of genes in the mitochondrial genome of terrestrial plants varies greatly and shows significant conservation, usually between 32 and 67. The size of mitochondria in plants is about 100-10,000 times that of animals. At present, the size of mitochondrial genomes of gramineous plants that have been assembled is also quite different (Hall et al., 2020; Tong et al., 2017; Li et al., 2025). In addition, there are significant differences in nucleotide substitution rate and repeat recombination level among different plant species. The characteristics of complete maternal inheritance also make it more accurate to study its evolution and interspecific differentiation. The data can be used to construct phylogenetic trees and determine the genetic relationship between species (Wu et al., 2022; Ou et al., 2024). However, so far, there has been no report on the mitochondrial genome of *Festuca pratensis*. Therefore, this study systematically assembled and annotated the first high-quality mitochondrial genome of *Festuca pratensis*, and analyzed its genomic structure and characteristics, repetitive sequences, codon preference analysis of protein-coding genes, prediction of RNA editing sites, mitochondrial sequences derived from chloroplasts, substitution rate of protein-coding genes, nucleotide polymorphisms, phylogenetic evolution and collinearity. These results are helpful for mining excellent genes of *Festuca pratensis* and provide data resources for its conservation biology, population genetics and evolutionary analysis.

## 2 Materials and methods

### 2.1 DNA extraction, genome sequencing, assembly and annotation of *Festuca pratensis*

Fresh young leaves of *Festuca pratensis* were collected from the National Perennial Forage Germplasm Resource Nursery located in Hohhot, Inner Mongolia, China (40.57°N, 111.93°E). Genomic DNA was extracted and purified using a modified CTAB method (Qiagen Blood & Cell Culture DNA Kit (Cat.no.13323)). The innovative third-generation Nanopore PromethION combined with the second-generation Illumina Novaseq6000 sequencing technology was used for library construction and sequencing. Finally, the PromethION platform sequencing obtained 31.43 GB raw data, and the Illumina platform sequencing obtained 35.54 Gb raw data. The Nanopore raw data obtained by sequencing were compared with the reference gene sequence (plant mitochondrial core gene, https://github.com/xul962464/plant_mt_ref_gene) using the software minimap2 (v2.1) (Sahlin et al., 2023) to obtain the mitochondrial genome sequence, and then corrected using the Canu software (Koren et al., 2017). Bowtie2 (v2.3.5.1) (Langmead and Salzberg, 2012) was used to align the second-generation data to the corrected sequence, and then use the Unicycler (v0.4.8) (Wick et al., 2017) comparison to stitch the second-generation data and the corrected third-generation data with default parameter. Finally, we use Bandage (v0.8.1) software (Wick et al., 2015) to visualize the stitching results and manually adjust.

*Festuca pratensis* mitogenomes were annotated using GeSeq (Tillich et al., 2017) with reference to previously released mitogenome data of *Festuca* species and then manually adjusting the data into a circular mitogenome model. The cp genome was annotated using Plastid Genome Annotator (PGA) tools (Qu et al., 2019). Subsequently, Geneious V9.0.2 was used to amend mistaken codons (Kearse et al., 2012). The genome map was visualized using the Organellar Genome Draw (OGDRAW) software (Greiner et al., 2019).

### 2.2 Repeat sequence identification

MISA v1.0 software (Beier et al., 2017) was used to identify SSR motifs in the mitochondrial genome of *Festuca pratensis*. The minimum number of repeats was set to mono-10, di-5, tri-4, tetra-3, penta-3, hexa-3, respectively. Tandem Repeats Finder (https://tandem.bu.edu/trf/trf.html) software (Benson, 1999) was used to identify tandem repeats. The Match value was 2, the Mismatch value was 7, the indel value was 7, the percent identity was 80, the PM was 10, the percent score was 50, and the maximum repeat length was 2000. REPuter software (Kurtz et al., 2001) was used to detect repeat sequences, and Hamming Distance 3 and E-value cutoff of 1 × 10^−5^ were used to divide them into forward, reverse, palindromic, or complementary repeats. The minimum number of repeats was set to 30.

### 2.3 Codon usage bias analysis

The RSCU values and amino acid composition of the protein-coding genes in the mitochondrial genome of *Festuca pratensis* were analyzed using codonW 1.4.4 software (Iriarte et al., 2021) according to the default parameters. Codon preferences were configured using Perl scripts. To select unique CDS, and plotted the results with R.

### 2.4 Prediction of RNA editing sites

The putative RNA editing sites were identified in mitochondrial PCGs by Deepred-Mt software (http://tubic.tju.edu.cn/Deepred-Mt/) with default parameter.

### 2.5 Chloroplast-derived mitochondrial sequence identification

The chloroplast genome data for F*estuca pratensis* were obtained from our assemblies. BlastN was used for homologous segments between the chloroplast and mitochondrial genomes with an E-value threshold of 1 × 10^−5^. TBtools-II v2.136 was employed to visualize the transfer of genes from chloroplasts to mitochondria (Chen et al., 2020).

### 2.6 Selective pressure calculation

Non-synonymous (Ka) and synonymous (Ks) substitution rates were calculated using KaKs_Calculator2 (Zhang, 2022), based on 25 protein-coding genes in *Festuca pratensis* versus 40 Poaceae species. The sequence alignment performed by MAFFT (version 7.131) (Katoh and Standley, 2013).

### 2.7 Nucleotide diversity analysis

Nucleotide diversity (pi) analysis was performed by DnaSP v6.12.01 (Rozas et al., 2017) between 33 common genes with 8 Poeae (JX999996.1, NC_072961.1, NC_077587.1, NC_082434.1, OZ173748.1, OZ174321.1, OZ243092.1, *Festuca pratensis*).

### 2.8 Phylogenetic analysis

Phylogenetic analysis was performed based on 25 common genes (*atp1*, *atp4*, *atp6*, *atp8*, *atp9*, *ccmB*, *ccmC*, *ccmFC*, *ccmFN*, *cox1*, *cox2*, *cox3*, *mttB*, *nad1*, *nad2*, *nad3*, *nad4*, *nad4L*, *nad5*, *nad7*, *nad9*, *rpl16*, *rps3*, *rps4*, *sdh4*) from 41 Poaceae species and 2 outgroups (*Arabidopsis thaliana* and *Glycine max*) (**Supplementary Table 1**). These two species were chosen due to their relatively distant phylogenetic relationship with the ingroup, well-characterized genomic resources, and common usage as outgroup taxa in relevant phylogenetic studies. The sequence of the reference accessions was download from the NCBI. The MAFFT program (Rozas et al., 2017) was used to perform multiple sequence alignment after PCGs sequence concatenated. The RAxML-NG v1.2.0 tool (Stamatakis, 2006) was used to analyze the sequence evolution model, and the optimal data model was selected as GTR + F + I + G, involving 1,000 rapid bootstrap resampling. The phylogenetic tree was constructed using the Bayesian method (MrBayes tool) (Huelsenbeck and Ronquist, 2001), and the sequence evolution model combination was set to HKY + F + I + G4.

### 2.9 Synteny

The mitochondrial genomes of eight Poaceae species (JX999996.1, NC_072961.1, NC_077587.1, NC_082434.1, OZ173748.1, OZ174321.1, OZ243092.1, *Festuca pratensis*), which are closely related to *Festuca pratensis*, were selected from the NCBI database for synteny analysis. Firstly, the homologous sequences of these eight species were pairwise compared using BlastN 2.13.0 (Altschul et al., 1990), and homologous sequences with a length of more than 300 bp were selected as collinear blocks. Subsequently, multicollinear maps were constructed using MCScanX (Wang et al., 2012) with block >300bp.

## 3 Results

### 3.1 Genomic features of the *Festuca pratensis* mitogenome

**Figure 1:**
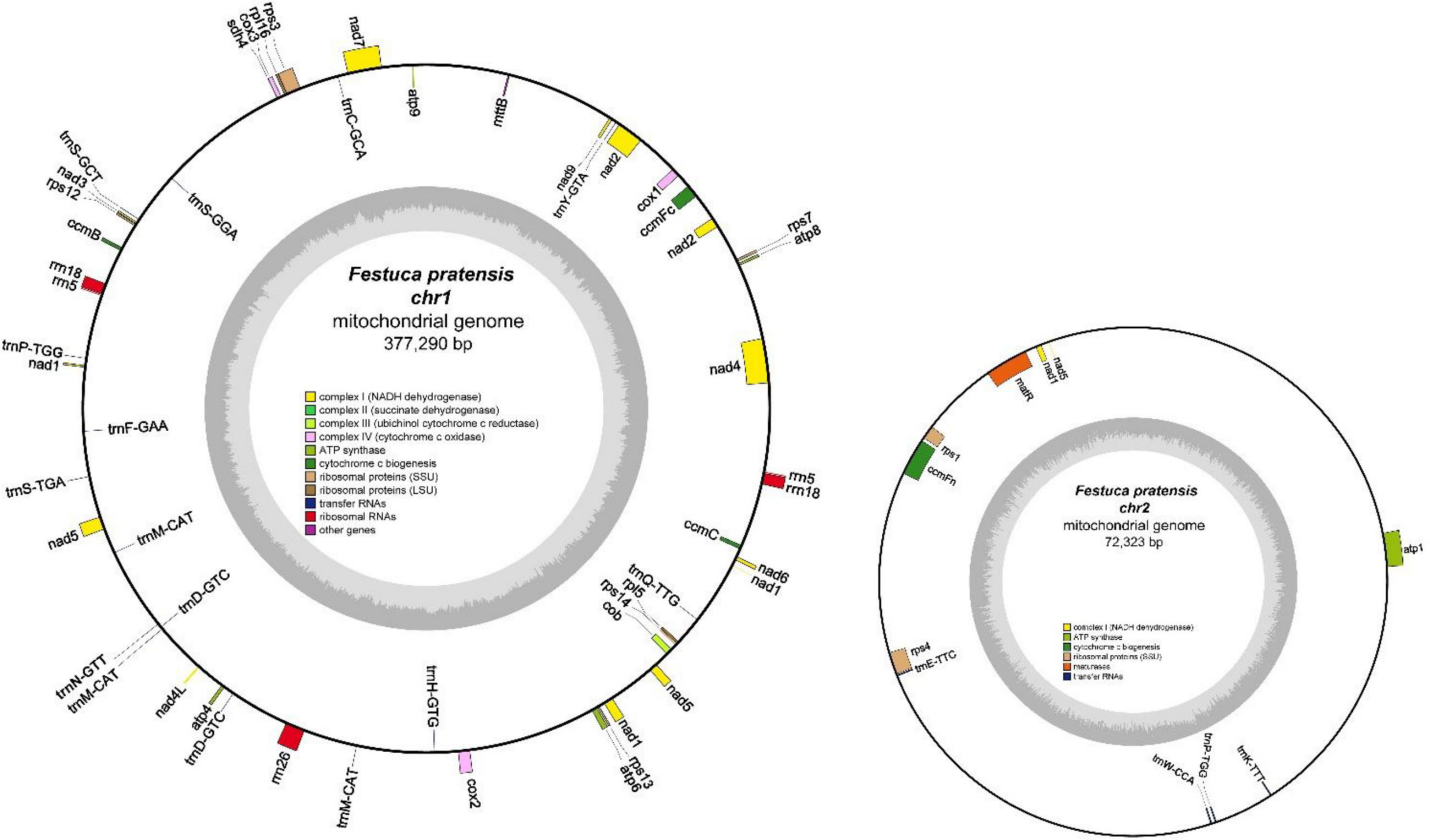
Circular representation of the complete mitochondrial genome of *Festuca pratensis*. Depicting a circular map, genes on the outer and inner rings are annotated to indicate forward and reverse transcription directions, respectively. The inner ring features a charcoal grey gradient representing GC content, while various functional gene groups are highlighted with distinct colors.

Unlike most plants, the mitochondrial genome of *Festuca pratensis* is a multichromosomal structure with a total length 449,613 bp (**Table 1**; **Figure 1**). The genome size of Chr1 is 5.22 times that of Chr2 (377,290 bp and 72,323 bp). The number of CDS is 32, and the number of tRNA and rRNA is 19 and 5, respectively (**Table 1**). Notably, rRNA and two pseudogenes were only identified in Chr1 (**Supplementary Table 2**). GC content refers to the percentage of guanine (G) and cytosine (C) bases in a DNA sequence. In this study, the GC contents of Chr1 and Chr2 were 44.42% and 44.30%, respectively. The specific genome assembly information is shown in **Table 1** and **Figure 1**. The datasets presented in this study can be found in online repositories. The names of the repository and accession number(s) can be found below: https://www.ncbi.nlm.nih.gov; Genbank accession number: PZ250838 and PZ250839.

**Table 1:** Specific mitogenome assembly information of *Festuca pratensis*.

Total mitochondrial genome	Total	Mitochondrial chromosome 1	Mitochondrial chromosome 2
Length (bp)	449613	377290	72323
GC content (%)	44.23	44.22	44.30
Number of CDS	32	27	5
Length of CDS (bp)	29430	22245	7185
Percentage of CDS (%)	6.55	5.90	9.93
CDS GC content (%)	42.72	41.71	45.83
Number of tRNA	19	15	4
Length of tRNA (bp)	1447	1151	296
Percentage of tRNA (%)	0.32	0.31	0.41
tRNA GC content (%)	51.14	51.78	48.65
Number of rRNA	5	5	/
Length of rRNA (bp)	7647	7647	/
Percentage of rRNA (%)	1.70	1.70	
rRNA GC content (%)	53.24	53.24	/
Number of pseudogenes	2	2	/

The mitochondrial genome of *Festuca pratensis* includes 51 genes, which can be divided into four categories according to different functions (**Supplementary Tables 2**-**4**). Among them, there are 14 core genes, 15 tRNA genes. The core genes are mainly involved in ATP synthase, Cytochrome c biogenesis, Ubiquinol cytochrome c reductase, Cytochrome c oxidase, Maturases and other processes; the tRNA gene is mainly related to RNA transport (**Supplementary Tables 2**-**4**). The number of variable genes was the largest (19) among the four types of genes, which were mainly related to NADH dehydrogenase, Ribosomal proteins (LSU), Ribosomal proteins (SSU), Succinate dehydrogenase and other functions (**Supplementary Tables 2**-**4**). The smallest number of rRNA genes (3), which are mainly involved in the Ribosomal RNAs process (**Supplementary Tables 2**-**4**). The intron number varies from 0 to 4, and only nad4 contains 3 introns (**Supplementary Tables 2**-**4**). Multi-copy genes were all distributed in functional RNA, and all of them were 2 copies except for *trnM-CAT*, mainly including *rrn5*, *rrn18*, *trnD-GTC* and *trnP-TGG* (**Supplementary Tables 2**-**4**). A total of two pseudogenes were detected, both of which were hypothetical protein-coding genes (*rps14* and *sdh4*) (**Supplementary Tables 2**-**4**).

### 3.2 Anatomization of repeat sequences

**Figure 2:**
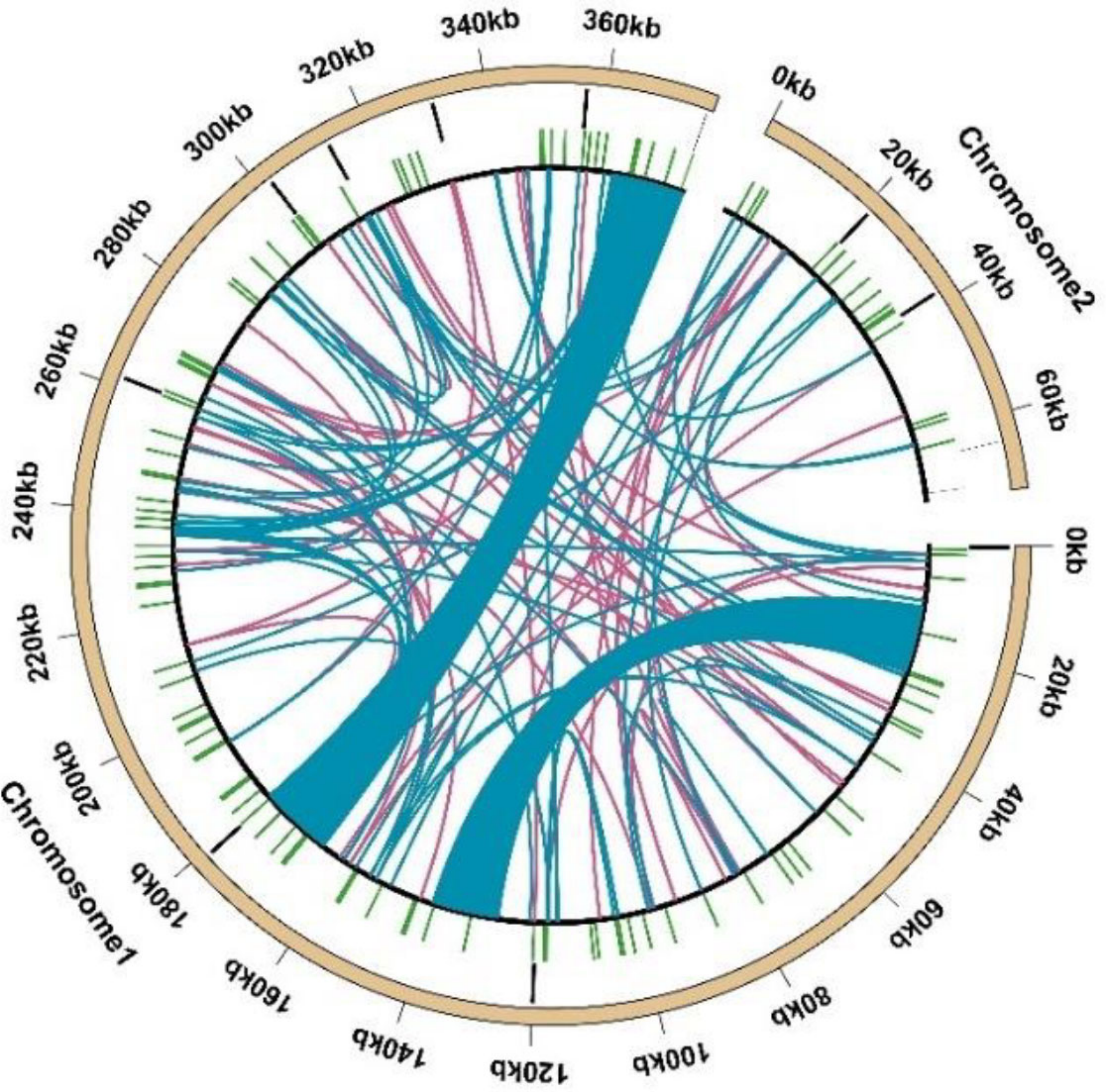
The distribution of dispersed repeats among the mitochondrial genome of *Festuca pratensis*. The outermost circle represents the mitochondrial (light brown) genome sequence, followed by tandem repeats (black), SSR repeats (green) and dispersed repeats (deep sky blue arcs represent forward repeats and the deep pinkish purple represents palindromic repeats).

**Figure 3:**
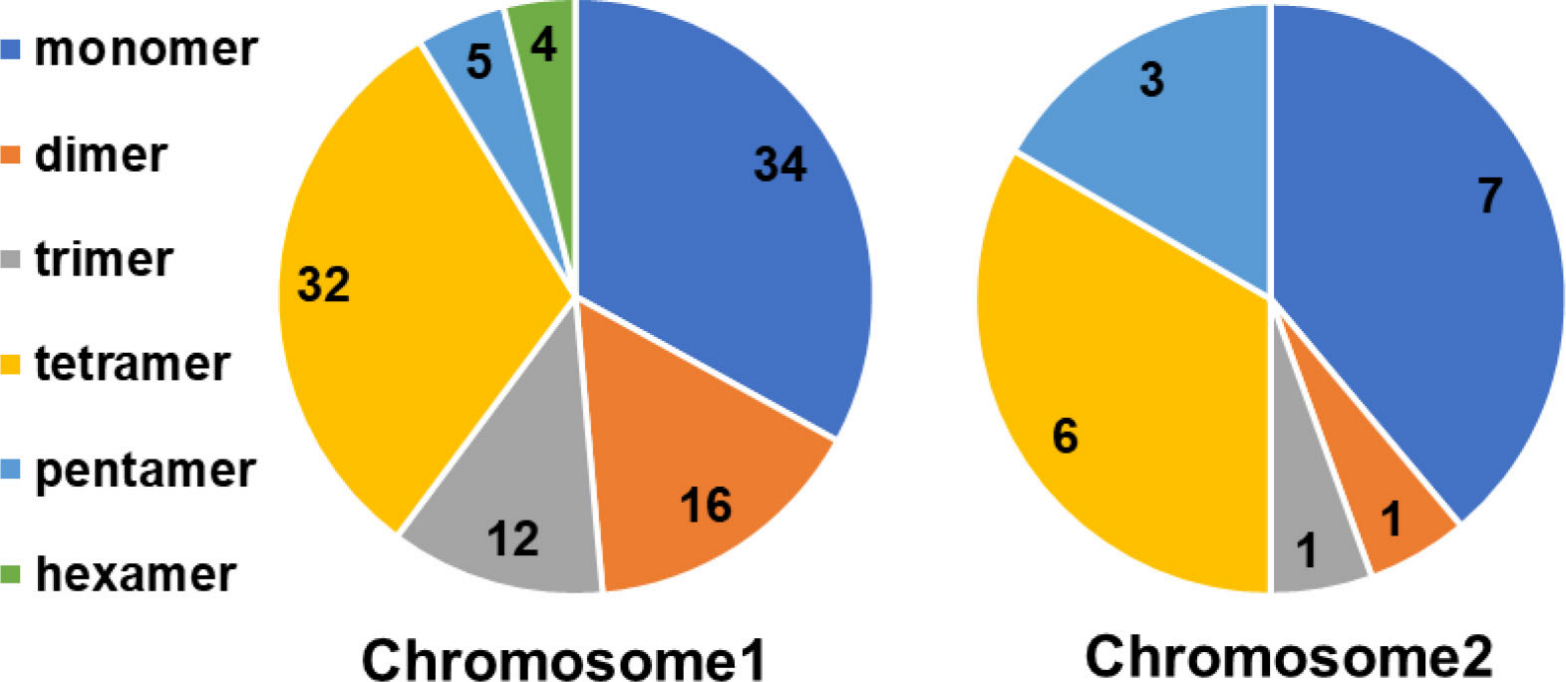
Distribution of SSRs in *Festuca pratensis* mitochondrial genome.

The mitochondrial genome of *Festuca pratensis* contains three types of repeats: simple repeats (121), tandem repeats (12), and scattered repeats (112) (**Figure 2**). The 121 simple repeat sequences were all perfect repeats, and no compound SSR was detected (**Supplementary Table 5**). A total of 103 SSR sequences were detected on Chr 1, which was nearly 6 times that of Chr 2 (**Figure 3**). Among the six nucleotide repeat types, mononucleotide and tetranucleotide repeat types accounted for the top two (33.88% and 31.40%), followed by dinucleotide (14.05%), trinucleotide (10.74%) and pentanucleotide (6.61%), respectively. It is worth noting that the distribution of hexanucleotide repeat types was not detected in Chr 2 (**Figure 3**). The 121 simple repeat sequences were located in the intergenic spacer (IGS) (**Supplementary Table 5**). Although they were not directly involved in protein synthesis, their polymorphism and regulatory potential could be the key to plant genome research. SSR sequence distribution was not detected in introns and gene coding regions (**Supplementary Table 6**). The single nucleotide repeat motif ‘ A ‘ on Chr1 had the largest number of repeats, while the minimum number of repeats (3) was as high as 47 (**Supplementary Table 6**). The number of repeat motifs of the remaining 74 SSR sequences was distributed between 4–14 times (**Supplementary Table 6**). 36 repeat types were detected in all SSR repeat sequences, of which A/T (40, 33.06%) repeat motifs were the most, followed by AG/CT (10, 8.26%), AAAG/CTTT (10, 8.26%), AATG/ATTC (10, 8.26%), and the remaining repeat motifs were less than 5 times (**Supplementary Table 6**).

**Figure 4:**
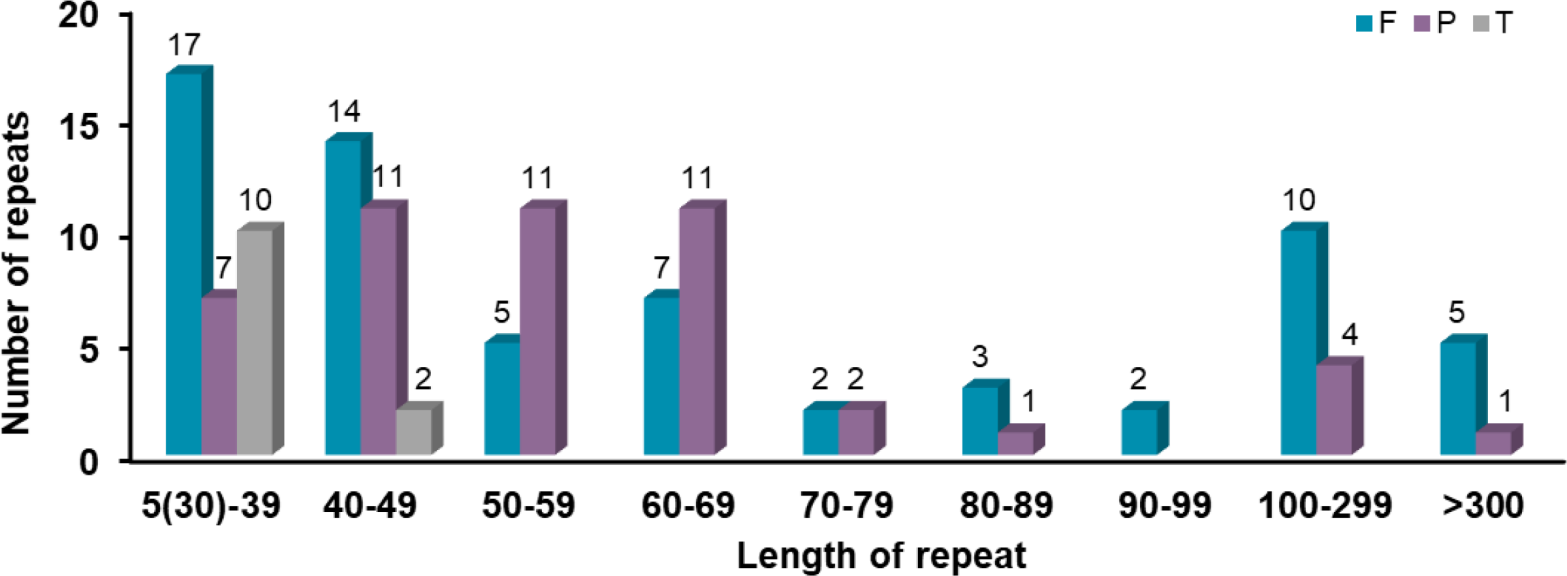
Allocation of the lengths of dispersed repeats in *Festuca pratensis* mitochondrial genome. The X-axis indicates the types of dispersed repeats and the ordinate indicates the number of scattered repeats.

Scattered repetitive sequences have been proved to be key players in genome function and evolution. In this study, a total of 112 scattered repetitive sequences were detected in the mitochondrial genome of *Festuca pratensis* (**Figure 2**). There were 64 forward repeats and 48 palindromic repeats (**Figure 2**). These two types of repeats were distributed on both chromosomes. The total length of all the scattered repetitive sequences was 38,651 bp, with an average length of 345.10 bp (**Supplementary Table 7**). Among them, there were two sequences with a length of more than 10000 bp, both of which were positive repetitive sequences, which were located in the gene coding region (*rrn18* and *rrn5*) and the intergenic region (*cox3*, *trnS-CGA*, *nad4* and *atp8*) (**Supplementary Table 7**). The longest palindromic repeat sequence was only 341 bp, encoding two intergenic spacer genes (*trnD-GUC*, *rrn26*, *trnM-CAU* and *atp4*), showing a huge difference from the forward repeat sequence (42.59 times) (**Supplementary Table 7**). Most of the scattered repeats were distributed below 100 bp (92, 82.14%) (**Figure 4**). In all the scattered repeat sequences, the proportion of some or all of them located in the IGS region was 95.54%, which was slightly lower than the proportion of simple repeat sequences in the intergenic region (100%) (**Supplementary Table 7**).

A total of 12 tandem repeats were detected in the complete mitochondrial genome of *Festuca pratensis* (**Figure 2**). Except for one gene encoding *atp6*, the rest were located in the intergenic spacer (IGS), which was consistent with the distribution of the above two types of repeats in the mitochondrial genome (**Supplementary Table 8**). The proportion of tandem repeats with an alignment rate of more than 95% was 91.67% (**Supplementary Table 8**). The length of 12 tandem repeats ranged from 5 to 43 bp, and the copy number variation ranged from 1.9 to 8.2 (**Supplementary Table 8**).

### 3.3 Codon usage analysis of PCGs

**Figure 5:**
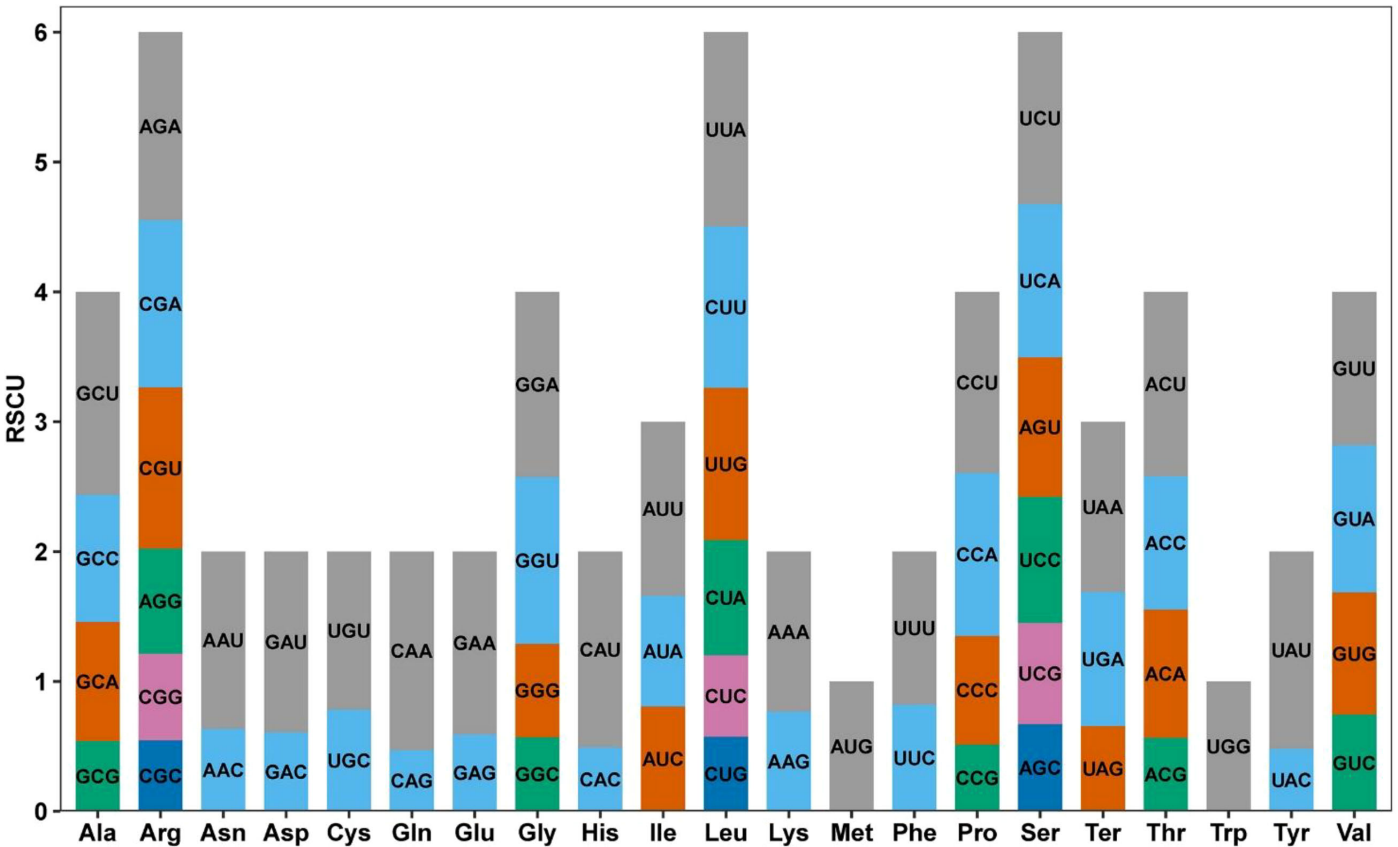
Relative synonymous codon usage (RSCU) in PCGs of *Festuca pratensis* mitochondrial genome. Codon families are on the abscissa.

A total of 9,810 codons encoding 33 PCGs were detected in the mitochondrial genome of *Festuca pratensis*, which were distributed in 21 amino acid families (**Figure 5**, **Supplementary Table 9**). The amino acids with the highest frequency of occurrence were Ile (766, 7.81%), Leu (1069, 10.90%) and Ser (860, 8.77%), and the lowest was Ter (32, 0.33%) (**Supplementary Table 9**). The number of occurrences of other amino acids ranged from 138 to 695 (**Supplementary Table 9**). The analysis of relative synonymous codon usage (RSCU) showed that the probability of codon preference and avoidance was 50% (RSCU >1) and 47% (RSCU <1), respectively (**Supplementary Table 9**). Another 3% of the codon RSCU was 1 (Met (AUG) and Trp (UGG)), indicating that its frequency of use was consistent with random expectations and had no preference (**Supplementary Table 9**). The most frequently used codon was Ala (alanine) GCU, and its RSCU was 1.5628 (**Supplementary Table 9**). The most avoided codon is Gln (glutamine) CAG, which RSCU is 0.4702 (**Supplementary Table 9**). One amino acid in plants usually corresponds to multiple codons, but the same amino acid has different preferences for different codons. For example, among the three codons (UAA, UGA and UAG) encoding Ter, the first two RSCUs are greater than 1, while the RSCU of UAG is only 0.6562 (**Supplementary Table 9**). In addition, the base endings of codons are usually related to tRNA abundance matching, genomic GC content, translation accuracy and other mechanisms. In this study, we prefer to use codons with A/U base endings and codons with G/C endings of 32 and 32, respectively (**Supplementary Table 9**).

### 3.4 RNA editing sites prediction

**Figure 6:**
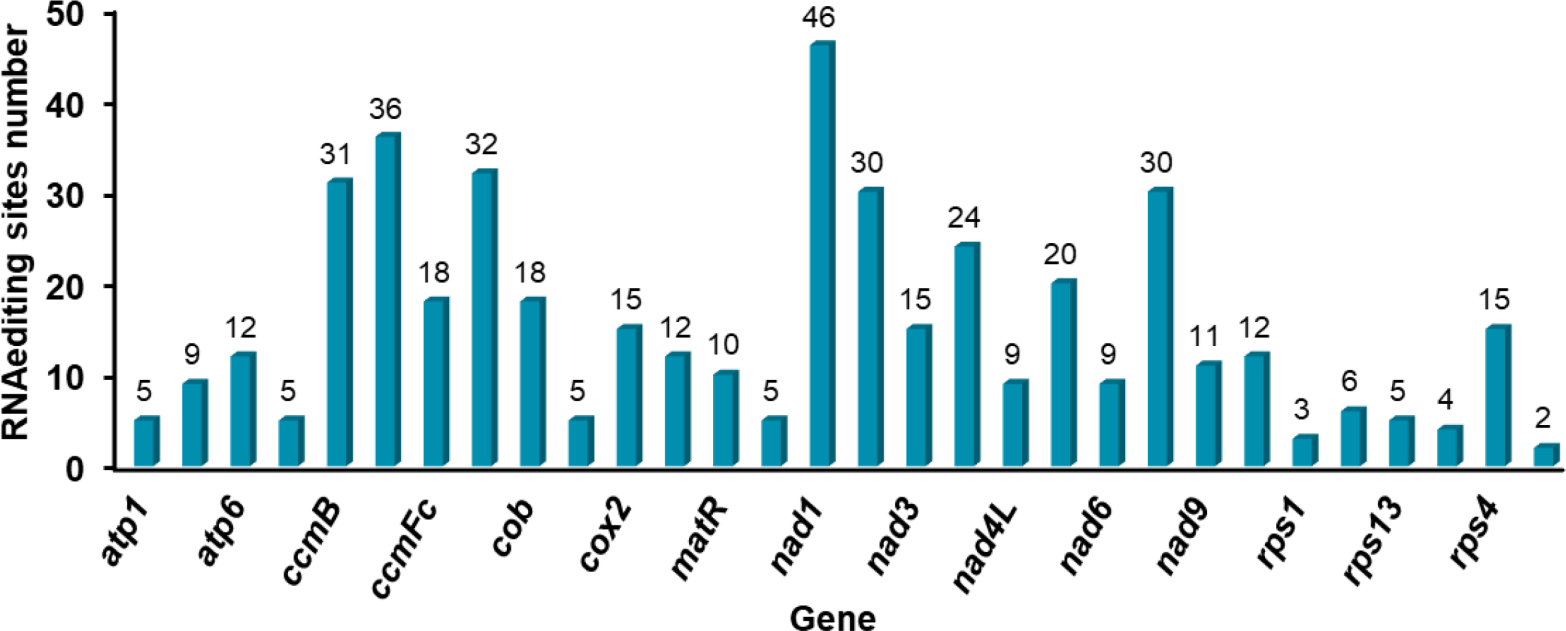
The distribution of RNA editing sites in the mt PCGs of *Festuca pratensis*.

**Figure 7:**
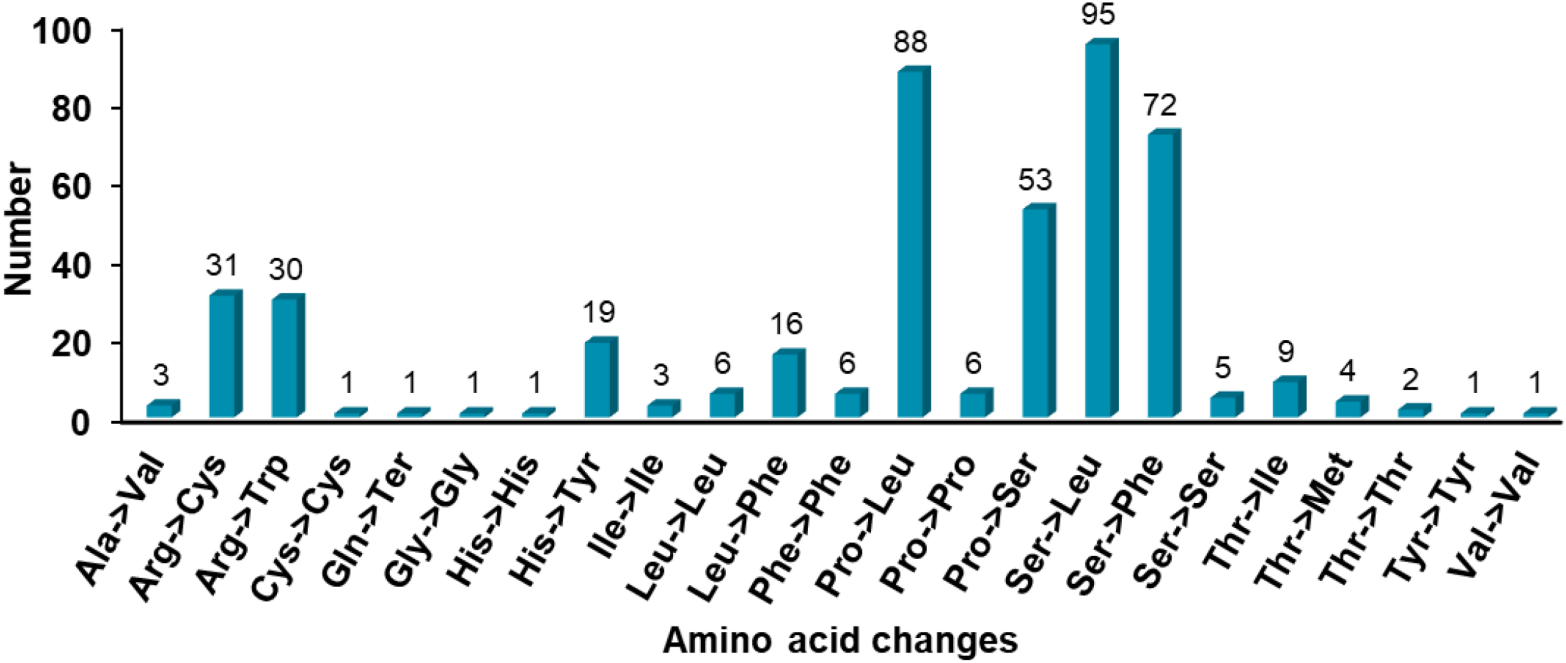
Frequency of amino acid changes caused by RNA editing of *Festuca pratensis* mitochondrial genome.

RNA editing events can increase protein diversity, correct genetic information, regulate gene expression, and often occur in plant organelles. In this study, a total of 454 effective RNA editing sites were detected in the mitochondrial genome of *Festuca pratensis*, distributed on 30 PCGs, accounting for 90.91% of the total PCGs, with an average of 15.13 change sites per gene (**Figure 6**; **Supplementary Table 10**). The *ccmC* (36) and *nad1* (46) genes had the most RNA editing sites, and the *rps7* (2) gene editing sites were the least (**Figure 6**; **Supplementary Table 10**). The number of editing sites detected in the remaining genes ranged from 3 to 32, respectively (**Figure 6**; **Supplementary Table 10**). In this study, only one type of base change, namely C-U change, was detected at all editing sites (**Supplementary Table 10**). Among the 56 codon changes, 26 (46.43%) occurred in the second base, followed by the first base (n = 17, 30.36%), and the third base (n = 13, 23.21%) had the least change (**Figure 7**; **Supplementary Table 10**). Eleven synonymous changes were detected in 23 amino acid change types, which may increase the genetic redundancy and fault tolerance of the mitochondrial genome of *Festuca pratensis*. Among them, the most common is Ser- > Leu (n = 95) conversion (**Figure 7**).

### 3.5 Chloroplast-derived mitogenomic sequences

**Figure 8:**
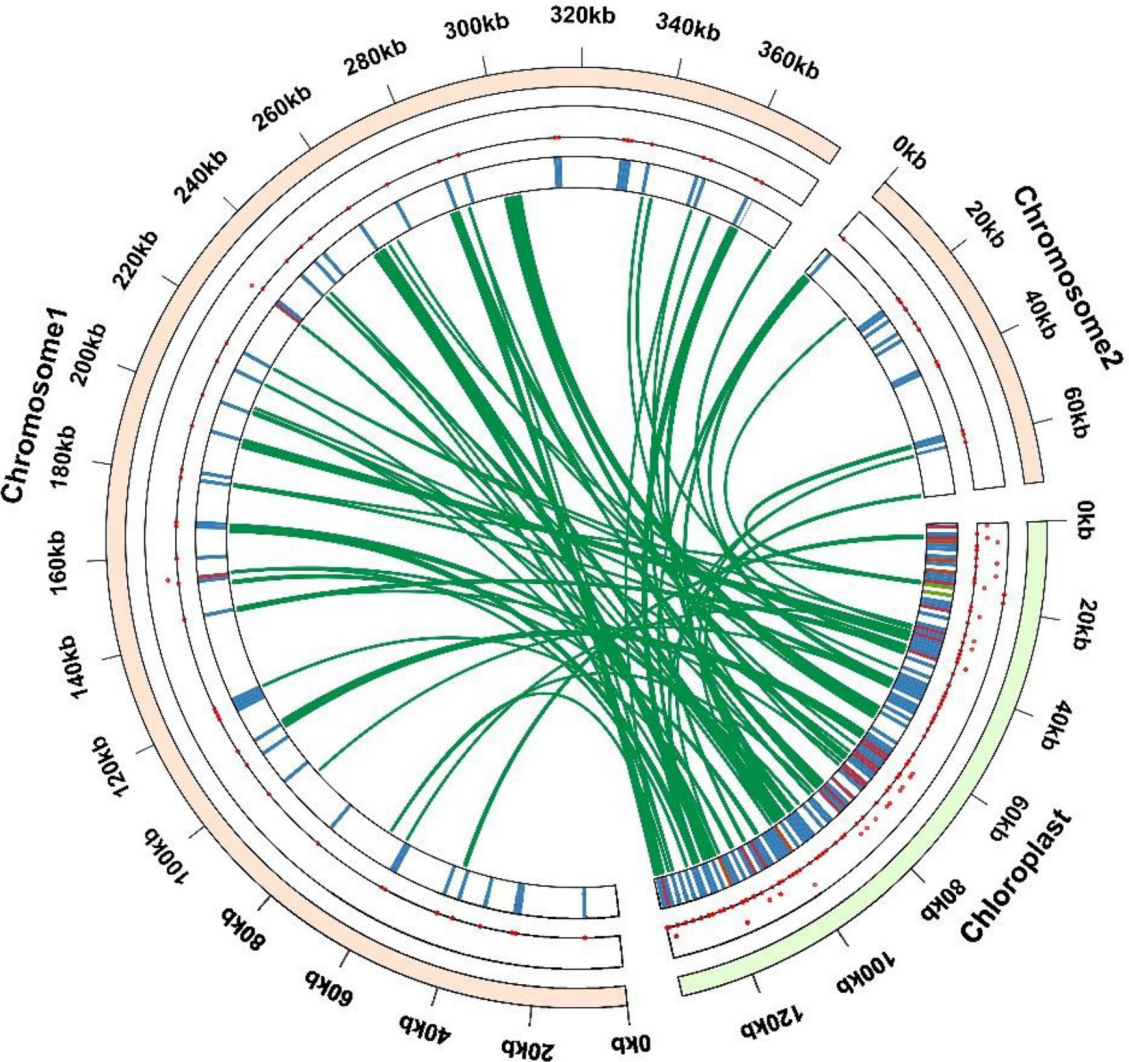
Gene transfer events between the chloroplast and mitochondrial genome. Dots maps inside the two chromosomes demonstrate where the migrated genes are located. The light-green circular segment represents the chloroplast genome, and the light brown circular segments depict the mitogenome. The green lines in the circle portray the routes of chloroplast-like sequences inserted from the cp genome into the mitogenome.

Gene exchange is often carried out among mitochondria, chloroplasts and nuclei in the process of biological evolution. This gene transfer is usually two-way, which is of great significance for plant hybrid breeding and heterosis utilization. In order to observe the gene flow between the mitochondrial genome and the chloroplast genome of *Festuca pratensis*, a total of 84 transfer fragments were identified (**Figure 8**; **Supplementary Table 11**). These fragments in the chloroplast genome were mainly rRNA and tRNA genes, and a few were protein-coding genes located in the intergenic region (**Supplementary Table 11**). The total length of the transfer fragment was 29,486 bp, accounting for 6.56% of the total length of the mitochondria of the *Festuca pratensis* (**Supplementary Table 11**). The total length of the transfer fragment of Chr 1 was 26,331 bp, and the total length of the transfer fragment of Chr 2 was 3,155 bp, which was consistent with the trend of the total length of the two chromosomes (**Supplementary Table 11**). A total of 4 completely transferred DNA fragments were found, but their matching degree did not reach 100%, which were rps12 (72.067), trnS-UGA (80.519), trnM-CAU (94.521) and trnN-GUU (96.739) (**Supplementary Table 11**). The remaining DNA fragments have undergone various forms of transfer between genes, intergenic regions, and introns, indicating that the communication between the two is more frequent. The longest transfer fragment was *rpoC1* in chloroplast, and *rpoC1* (intron) was transferred to mitochondrial IGS (*cox2*, *atp6*) (**Supplementary Table 11**). The shortest transfer fragment was *atpF* in chloroplast transferred to mitochondrial IGS (*mttB*, *atp9*) (**Supplementary Table 11**). The transfer frequency can show the rate of biological evolution and the selection advantage. The DNA fragment with the highest evolution frequency in this study was *rrn16* in chloroplast to *rrn18* and IGS (*rrn18*, *rrn18*) in mitochondria (16 times) (**Supplementary Table 11**). A total of 12 fragments were found by counting all fragments of mitochondrial transport and maintaining the integrity of most fragments (**Supplementary Table 11**). Including 8 tRNA genes (*trnF-GAA*, *trnH-GUG*, *trnK-UUU*, *trnM-CAU*, *trnN-GUU*, *trnP-UGG*, *trnS-GGA*, *trnS-UGA*), 2 rRNA genes (*rrn18* and *rrn26*) and 2 PCGs (*atp1* and *rps12*) (**Supplementary Table 11**).

### 3.6 Ka/Ks

**Figure 9:**
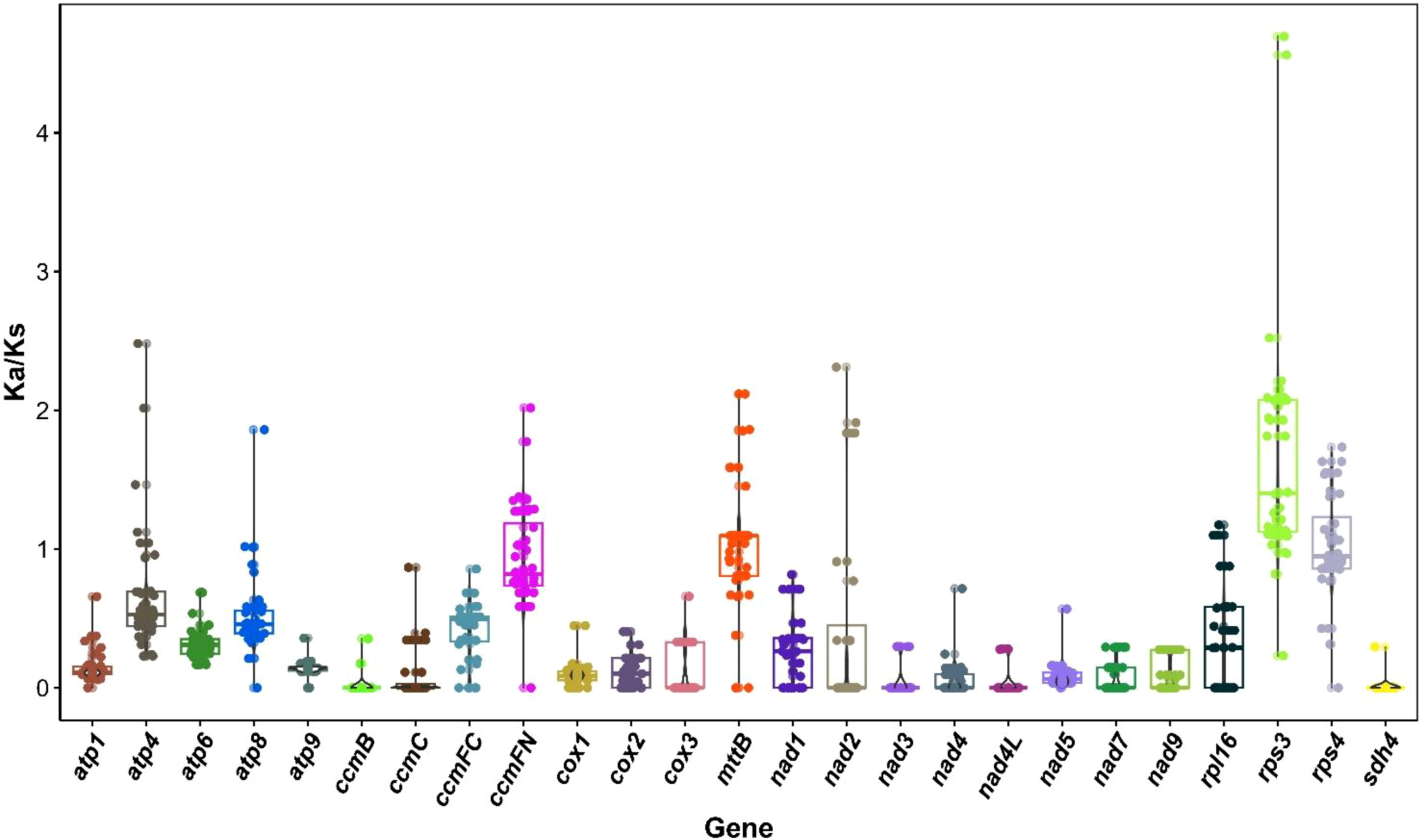
The Ka/Ks values of 25 protein-coding genes in *Festuca pratensis* versus 40 Poaceae species.

The substitution rates of 25 PCGs related to the mitochondrial genome of *Festuca pratensis* were analyzed in 40 gramineous species (**Figure 9**; **Supplementary Table 12**). It was found that these 25 PCGs had different degrees of deletion in different species. The top three species with the largest number of deletions were *Lolium perenne* (17), *Hierochloe odorata* (9), *Avena longiglumis* (8), *Aegilops tauschii* (8) and *Poa chaixii* (8) (**Figure 9**). The top three species with the least missing number were *Zea mays* subsp.*Mays* (1), *Zea perennis* (1) and *Zea luxurians* (1) (**Figure 9**). Only one PCGs of *Eleusine indica*, *Polypogon viridis* and *Tripidium rufipilum* had Ka/Ks > 1, and the rest were missing or < 1, indicating that these three sequences were more affected by negative selection during evolution to maintain the conserved function of proteins (**Supplementary Table 12**). The *rps3* gene showed positive selection (Ka/Ks > 1) in 36 (90%) of the 40 species, indicating that the amino acid changes favorable for biological adaptation were retained, and it may be a functional gene adapted to rapid evolution (**Supplementary Table 12**). Genes such as *ccmC*, *nad4*, and *nad7* have Ka/Ks = 0 in most species, with ratios of 69.70%, 64.70%, and 58.97%, respectively (**Supplementary Table 12**). These genes may be conserved sequences in evolution and are not subject to significant selection pressure.

### 3.7 Nucleotide diversity analysis

**Figure 10:**
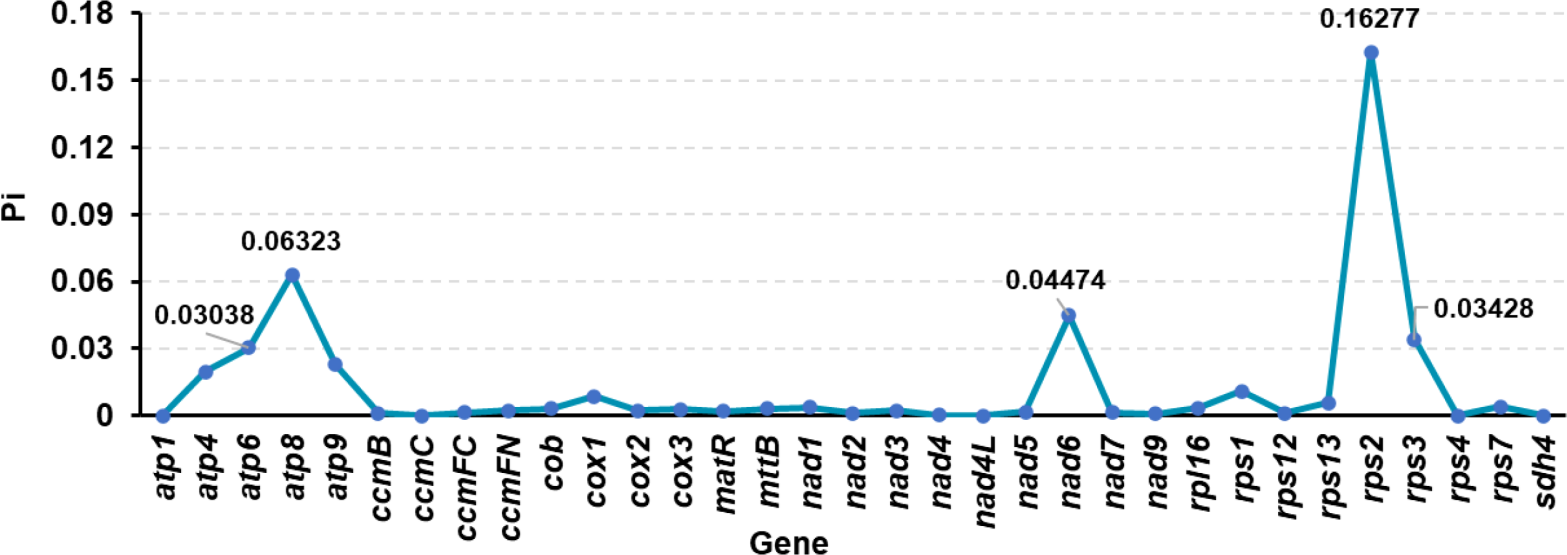
Nucleotide diversity of 8 Poaceae species (33 common genes).

Nucleotide polymorphism analysis is the basis for analyzing population genetic diversity and evolutionary dynamics. Based on the synonymous substitution rate analysis, we performed nucleotide polymorphism analysis on 33 PCGs shared by 8 Poaceae species to explore the selection variation of genes in the mitochondrial genome of *Festuca pratensis* under different selection pressures (**Figure 10**). The Pi values of 33 genes ranged from 0 to 0.16277 (**Figure 10**).

### 3.8 Phylogenetic analysis

**Figure 11:**
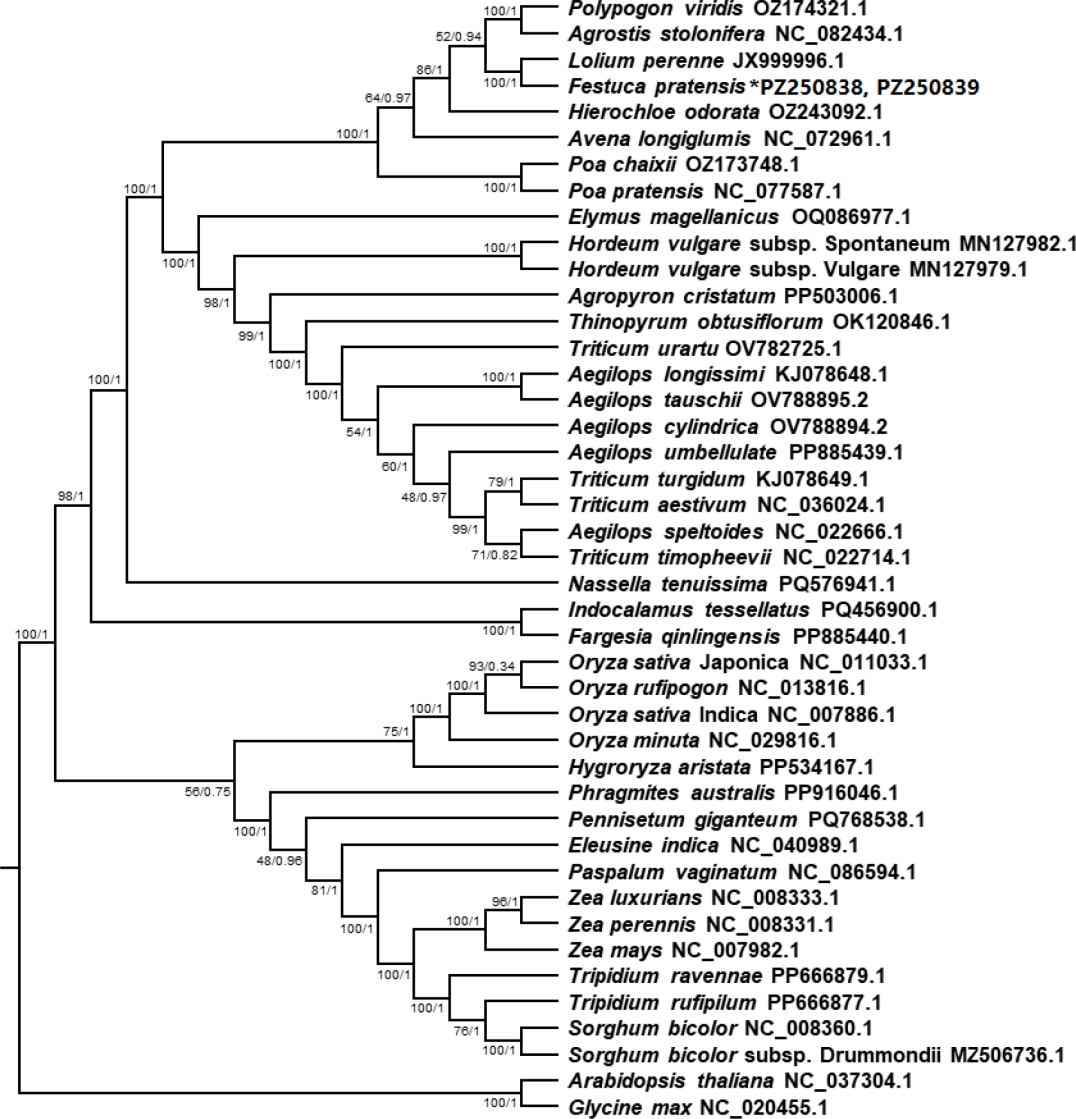
The phylogenetic relationships of *Festuca pratensis* compared with that of 40 Poaceae species. The ML bootstrap support values and Bayesian posterior probabilities are shown for each node.

The phylogenetic tree was constructed by using the mitochondrial genome data of 40 published Poaceae species, the mitochondrial genome data of *Festuca pratensis* obtained by sequencing in this study and the mitochondrial genomes of two leguminous plants to explore the evolutionary characteristics of the mitochondrial genome of *Festuca pratensis* in a large number of populations (**Figure 11**). The 43 species can be divided into 6 categories (**Figure 11**). The first category includes 8 species, mainly composed of turf grasses such as *Polypogon viridis* and *Agrostis stolonifera*, and also includes the *Festuca pratensis* in this study, which has the closest relationship with *Lolium perenne* (**Figure 11**). The second category includes 14 species, mainly composed of *Hordeum*, *Triticum* and *Aegilops* (**Figure 11**). The third category includes one species *Nassella tenuissima*; the fourth category includes two species, *Indocalamus tessellatus* and *Fargesia qinlingensis* (**Figure 11**). The fifth category includes 16 species, which are mainly composed of *Oryza*, *Zea* and *Sorghum* (**Figure 11**). The last category is two aliens, and the phylogenetic tree clearly divides 43 species into Leguminosae and Poaceae (**Figure 11**). Among the 41 nodes of the phylogenetic tree, there were 22 nodes with ML bootstrap support values of 100, accounting for 53.66% of all nodes, indicating that the results of this study were reliable (**Figure 11**). The results of phylogenetic tree also showed that *Festuca pratensis* had a distant relationship with *Oryza*, *Zea*, *Sorghum*, and had a close relationship with *Triticum*, *Aegilops*, *Poa*, *Lolium* and other grasses, indicating that *Festuca pratensis* may experience less artificial domestication process, which was consistent with other conclusions of our study (**Figure 11**). At the same time, it is proved that the mitochondrial genome can be effectively used for phylogenetic analysis of species and exploration of their genetic relationship.

### 3.9 Synteny

**Figure 12:**
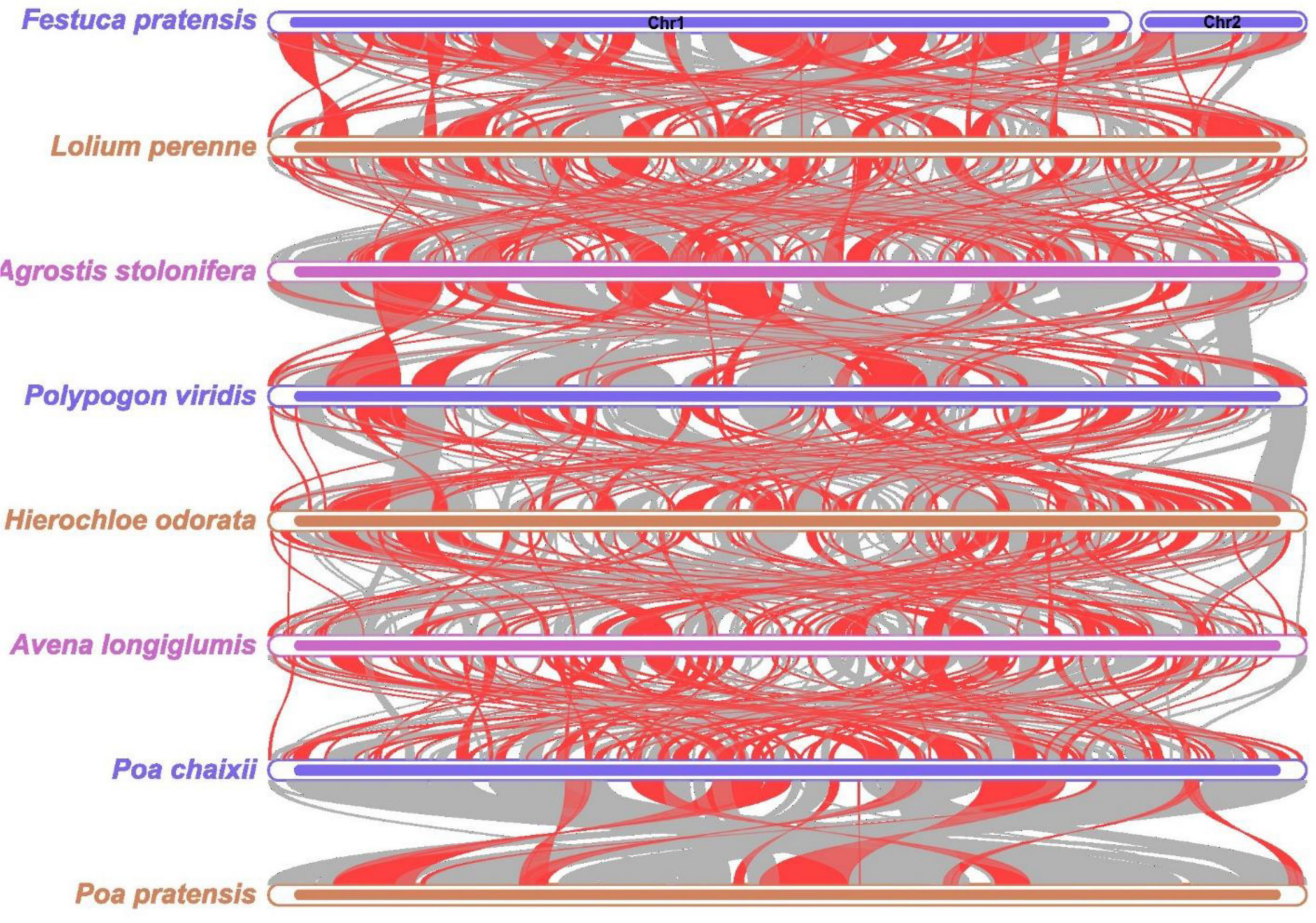
Synteny analysis of eight Poaceae species. Red-curved regions indicate where inversions occur, gray regions indicate regions of good homology, and white regions indicate species-unique sequences.

The collinearity analysis of the mitochondrial genomes of 8 Poaceae species (including *Festuca pratensis*) showed that there were significant differences in the proportion of collinearity among different species (**Figure 12**; **Supplementary Table 13**). The proportion of collinearity fragments in the reference genome (Prop in ref) was 0.5527-0.8756, and the proportion in the query genome (Prop in query) was 0.5852-0.9333 (**Supplementary Table 13**). Specifically, the degree of collinearity between *Poa chaixii* and *Poa pratensis* is the highest, and the proportion of collinearity in the query genome *Poa pratensis* is as high as 0.9333, which indicates that the genome structure of the two is highly conserved and the genetic relationship may be closer (**Supplementary Table 13**). In contrast, the proportion of collinearity between *Hierochloe odorata* and *Avena longiglumis* was relatively low (0.5765), indicating that these two species may have experienced more genome rearrangement events during evolution (**Supplementary Table 13**).

## 4 Discussion

Mitochondria are called cell ‘energy factory’, which belong to semi-autonomous organelles and have independent genomes. Mitochondrial genome has the characteristics of maternal inheritance, high mutation rate, multiple copies and heterogeneity. It plays an important role in species identification, classification and phylogenetic evolution, population genetics and germplasm resource traceability, cytoplasmic male sterility and crop breeding, comparative genome and organelle evolution (Tian et al., 2021; Nitsch et al., 2024). *Festuca pratensis* is a perennial herb of the genus *Festuca*, Poaceae. It is often used for close hybridization with *Lolium perenne*. Its cytoplasmic male sterility (CMS) -related mitochondrial gene is an important research target for forage hybrid seed production (Majka et al., 2018). However, there is no report on the assembly of high-quality mitochondrial genomes of *Festuca pratensis*. The mitochondrial genome of Poaceae plants is extremely large and its structure is variable. Frequent homologous recombination between repeats leads to the production of a variety of structural isomers. At the same time, there are problems such as vulnerability to nuclear genome NUMT contamination and difficulty in extracting pure DNA (Zhu et al., 2025). Based on this, this study used the innovative third-generation Nanopore PromethION combined with the second-generation Illumina Novaseq6000 sequencing technology for library construction and sequencing, and obtained a high-quality *Festuca pratensis* mitochondrial genome with a total length of 449,613 bp, which provides new insights into the unique properties of the genus (**Figure 1**).

Mitochondrial genomes of Poaceae range in size from 321 kb (*Zizania latifolia*) to 704 kb (*Trichosanthes kirilowii*), showing the potential of mitochondrial genomes in genetic evolution, functional gene mining and structural variation (Gualberto and Newton, 2017). The plant mitochondria are large but usually contain only 50–60 coding genes. The coding region usually accounts for 7%-17% of the genome. The percentage of coding gene length in this study accounts for only 6.5% of the total mitochondrial genome length (**Supplementary Table 3**). There are 33 coding genes, which is consistent with the results of *Salix suchowensis* (Ye et al., 2017; Ong and Palmer, 2006), indicating that the difference in plant mitochondrial genome size is mainly caused by the expansion and contraction of non-coding sequences, rather than the change in the number of functional genes. Although the mitochondrial genome size is significantly different and the structural rearrangement is frequent, its functional genes remain highly conserved as a whole, which is crucial for the study of cytoplasmic male sterility (CMS) -related mitochondrial genes with stable functions (Chang et al., 2023).

In *Festuca* plants, the gene composition of the mitochondrial genome also reflects the typical characteristics of mitochondrial evolution in angiosperms. As shown in **Supplementary Table 3**, the core functional genes (such as *atp*, *co*x, *cob*, *nad* family) exist in the mitochondrial genome of *Festuca pratensis*, and only introns (*) are detected in some genes (such as *cox2*, *nad1*, *nad2*, etc.), indicating that the core function of energy metabolism remains highly conserved during evolution. However, in ribosomal protein genes, *rps14* and *sdh4* were identified as pseudogenes (#), which is similar to the trend of gene loss and pseudogenization observed in gramineous plants (Hall et al., 2020). During the evolution of angiosperms, the transfer of ribosomal protein genes (such as *rps14*, *rps1*, *rps12*, etc.) to the nuclear genome is a common event. When homologous genes in mitochondria lose their functional necessity, they may be pseudogenized due to mutation accumulation or lack of regulatory elements (Figueroa et al., 1999). The existence of two pseudogenes in *Festuca pratensis* implies that their functions may have been replaced by homologous copies in the nuclear genome, which is similar to the formation mechanism of *sdh4* pseudogenes in rice, that is, gene transfer is the main driving force for the formation of relatively complete but non-functional pseudogenes in mitochondria (Wang et al., 2024). In addition, the inherent high mutation rate and functional redundancy of the mitochondrial genome may also accelerate the pseudogenization process of genes such as *rps14* and *sdh4*. The formation of these pseudogenes not only reflects the dynamic evolution of the mitochondrial genome of *Festuca pratensis*, but also provides important clues for understanding the adaptive advantages of mitochondrial gene transfer to nuclear genome in grasses.

RNA editing sites are specific sites for base insertion, deletion or replacement after RNA transcription, which can change genetic information and produce protein diversity (Khosravi and Jantsch, 2021). The synonymous mutation rate of RNA editing sites in the mitochondrial genome of *Festuca pratensis* is only 4.19%, which is lower than that of *Liriodendron tulipifera*, indicating that the mitochondrial genome has potential in efficient molecular markers for species differentiation, migration, population history and other research (**Supplementary Table 10**) (Richardson et al., 2013; Edera et al., 2018). RNA editing sites often show obvious preference during evolution. In this study, the conversion from serine to leucine (20.93%) is often shown (**Supplementary Table 10**). This conversion is an important mutation in adaptive evolution by enhancing protein hydrophobicity and membrane stability and improving mitochondrial respiratory chain function efficiency (Li et al., 2024). Effective RNA editing sites were detected in 90.91% of PCGs, of which *nad1* gene had the most RNA editing sites (**Supplementary Table 10**). The possible reason was that its gene trans-splicing was complex, encoding the core functional subunit of complex I and was strongly evolutionary selected. It plays an important role in ensuring the normal function of complex I assembly and respiratory chain, and plays an important role in plant environmental adaptation. In recent years, a large number of studies have shown that plant mitochondrial genome RNA editing events play an important role in response to biotic/abiotic stresses (Tang and Luo, 2018; Vats et al., 2019; Mestanza et al., 2025), and also provide new thinking for exploring plant stress resistance genes in *Festuca pratensis*.

Repetitive sequences, known as the “dynamic skeleton” of mitochondrial DNA, play an important role in regulating replication and transcription, mediating recombination and rearrangement, shaping structural stability, and driving evolutionary adaptation (Dong et al., 2018). Three types of repeat sequences were identified in the mitochondrial genome of *Festuca pratensis*, including simple repeats (121), tandem repeats (12) and scattered repeats (112) (**Figure 2**). The SSR sequence detected in Chr 1 was nearly 6 times that in Chr 2, which may be related to the genome size of the two chromosomes (**Figure 2**). The proportion of mononucleotide and tetranucleotide repeat types was the highest, which was consistent with the research results in *Eleusine indica* (Hall et al., 2020). It may be because the single nucleotide repeat replication slip is the most likely to occur, the selection pressure is the smallest, and the A/T preference becomes the absolute dominant, which is consistent with the results of our study. In addition, 112 scattered repeats and 12 tandem repeats were detected, which could provide effective reference molecular markers for diversity evaluation and species identification of *Festuca pratensis* (**Figure 2**).

The codon is a translation unit composed of three bases on mRNA, which can determine the amino acid sequence. Its core function is to encode a respiratory chain protein and maintain cellular energy metabolism (Li et al., 2023). In this study, 50% and 47% of the codons were preferred or avoided, indicating that most codons evolved in the adaptive selection of *Festuca pratensis* (**Supplementary Table 9**). Codon usage bias affects translation efficiency, protein structure and functional stability, which may be related to the adaptation and selection process of organisms in long-term evolution (Feng et al., 2024). In this study, the most frequently used codon is Ala GCU, which is consistent with the results of *Graptopetalum Paraguayense* (**Supplementary Table 9**) (Zhou et al., 2026). This may be due to its optimal swing pairing with the mitochondrial tRNA Ala anticodon, higher translation efficiency or GCU synonymous substitution has little effect on protein structure and function, and is weakly subjected to purification selection pressure. In addition, the majority of codons in this study preferred to use codons ending in A/U bases (93.33%) (**Supplementary Table 9**). This may be due to the high A + T base composition bias of the mitochondrial genome of *Festuca pratensis*, which drives the third position of the codon to A/U enrichment or the codon ending in A/U is more matched with the mitochondrial tRNA swing pairing, which is more efficient in translation and more suitable for the needs of efficient expression of respiratory chain proteins.

Ka/Ks analysis quantifies the natural selection pressure acting on the evolution of coding genes. This study found that 25 PCGs present in *Festuca pratensis* exhibited varying degrees of gene loss among 40 gramineous species (**Figure 9**; **Supplementary Table 12**). *Lolium perenne* showed the highest number of missing genes (17), whereas only one gene loss was detected in each of *Zea mays* subsp. *mays*, *Zea perennis*, and *Zea luxurians* (**Supplementary Table 12**). The *rps3* gene has been regarded as an evolutionary ‘hotspot gene’ owing to its multifunctional roles in core translation regulation, DNA repair, and immune regulation. To define positive selection in this study, a threshold of Ka/Ks > 1 was adopted. Under this criterion, global strong purifying selection was maintained to preserve fundamental biological functions, while key functional domains or sites underwent continuous positive selection in response to environmental stress, host–pathogen interactions, and optimization of metabolism and translation. Significant positive selection signals were detected in most species (Ran et al., 2010; Yildiz and Ozkilinc, 2021). In accordance with the threshold above, 36 out of 40 gramineous species (90%) exhibited a Ka/Ks ratio > 1 in the *rps3* gene, indicating pervasive positive selection (**Supplementary Table 12**). This widespread positive selection may be closely associated with the adaptive evolution of *rps3* underlying its multiple functions, including efficient translation regulation, accurate DNA damage repair, and enhanced environmental adaptability in grasses. The nucleotide polymorphism Pi values of 33 genes were between 0 and 0.16277, showing obvious inter-gene differences as a whole, reflecting the heterogeneity of selection pressure on different genes during evolution (**Supplementary Table 12**). It not only reflects the high conservation of mitochondrial core functional genes, but also suggests that some genes may play a more diverse evolutionary role in species adaptation and evolution.

One of the important characteristics of mitochondrial genome evolution is its Intracellular Gene Transfer (IGT) between different organelle genomes, which can dynamically regulate the mitochondrial genome by increasing genomic complexity and structural diversity (Najer et al., 2024). A total of 84 transfer fragments were identified in *Festuca pratensis* (**Figure 8**; **Supplementary Table 11**). These fragments in the chloroplast genome are mainly rRNA and tRNA genes, accounting for 6.56% of the total length of the mitochondrial genome of *Festuca pratensis*, which is consistent with the results of *Bromus inermis* (**Supplementary Table 11**) (Sloan and Wu, 2014; Feng et al., 2025). A total of 4 completely transferred DNA fragments were found, which were *rps12*, *trnS-UGA*, *trnM-CAU* and trnN-GUU, indicating that there is continuous genetic material transfer between the nuclear genome and the mitochondrial genome (**Supplementary Table 11**). Although these fragments are intact, whether they have transcriptional and translational activities and specific functions still need to be verified by experiments. This study found that the most frequent DNA fragment transfer event was the transfer of the chloroplast-derived *rrn16* sequence to the mitochondrial genome (**Supplementary Table 11**). A total of 16 times were detected, and the transfer products were mainly *rrn18* in mitochondria and intergenic spacer (IGS, *rrn18*, *rrn18*), indicating that the fragment had a high ‘ transferability ‘ or the advantage of being selected for retention during evolution, suggesting that these regions may be hot spots for exogenous DNA integration, providing a new perspective for understanding the co-evolution of plant organelle genomes (**Supplementary Table 11**).

*Festuca pratensis* belongs to *Fescue*, which is the main representative of forage grass and ecological restoration grass species in China. *Festuca pratensis* is an important model plant for studying the evolution and early evolution of complex monocotyledonous plants. The accuracy of early classification of Poaceae species based on morphology has been questioned for a long time. In the early classification system, there are significant differences in the definition and division of the category and coverage of the family, and the classification unit division is also relatively broad and rough. In this study, the phylogenetic tree was constructed based on the mitochondrial genome data of 40 published gramineous species, the mitochondrial genome data of *Festuca pratensis* obtained by sequencing in this study and the mitochondrial genomes of two *Leguminous* plants (**Figure 11**). The above species were divided into six categories (**Figure 11**). Interestingly, *Festuca pratensis* has the closest genetic relationship with *Lolium perenne*. Early gramineous classifications such as Clayton and Renvoize (1986) and Tzvelev (1989) have pointed out that *Lolium* and *Festuca* are closely related. According to the Journal of Systematics and Evolution/World Checklist of Poaceae, they are classified into *Pooideae*, *Poeae* and *Loliinae* (Soreng et al., 2015; Soreng et al., 2017; Soreng et al., 2022). Molecular systematics confirmed that the genus Lolium is not monophyletic, but nested within the Schedonorus of the genus *Festuca* (Catalán et al., 2004, 2012); *Festuca pratensis* is the core diploid species of this group, forming a sister branch with *Lolium perenne*. The two are the most commonly used hybrid combinations in forage breeding, which are used to integrate the high quality and grazing resistance of ryegrass and the cold and disease resistance of *Festuca pratensis*. The high fertility and genetic stability of the hybrid offspring proved that the reproductive isolation was extremely weak and the genome was highly compatible, which also indicated the accuracy of this study.

## 5 Conclusions

In this study, the mitochondrial genome of *Festuca pratensis* was sequenced by second-generation and third-generation sequencing technology for the first time, and the first high-quality mitochondrial genome was assembled and annotated. The obtained mitochondrial genome is 449,613 bp in length and is a multichromosomal structure. A total of 51 genes were included, including 14 core genes, 15 tRNA genes, 19 variable genes, and 3 rRNA genes. The repetitive sequences, codon preference, RNA editing sites, Ka/Ks and Pi were comprehensively analyzed in order to analyze the structural characteristics of the mitochondrial genome, codon usage patterns, post-transcriptional regulation, selection pressure characteristics and genetic diversity level. A total of 84 gene transfer fragments were found between the mitochondrial genome and the chloroplast genome, indicating that there are frequent genetic material exchange and recombination events between the plastid-mitochondrial genome. Phylogenetic analysis and collinearity analysis showed that there was a close relationship between *Festuca pratensis* and *Lolium perenne*, which was consistent with the results of morphological identification. It further proved the accuracy, integrity and scientific reliability of the mitochondrial genome assembled in this study. At the same time, it also confirmed the feasibility of phylogenetic classification and genetic relationship analysis of Poaceae forages based on mitochondrial genome sequences, which provided high-quality and reliable reference genome data for subsequent molecular evolution among closely related species, co-evolution of organelle genomes and genetic relationship identification of germplasm resources.

## Data Availability

The datasets presented in this study can be found in online repositories. The names of the repository/repositories and accession number(s) can be found in the article/**Supplementary Material**.
